# Nurses and Night Shifts: Poor Sleep Quality Exacerbates Psychomotor Performance

**DOI:** 10.3389/fnins.2020.579938

**Published:** 2020-10-14

**Authors:** Marco Di Muzio, Giulia Diella, Emanuele Di Simone, Luana Novelli, Valentina Alfonsi, Serena Scarpelli, Ludovica Annarumma, Federico Salfi, Mariella Pazzaglia, Anna Maria Giannini, Luigi De Gennaro

**Affiliations:** ^1^Department of Clinical and Molecular Medicine, Sapienza University of Rome, Rome, Italy; ^2^Department of Biomedicine and Prevention, University of Rome Tor Vergata, Rome, Italy; ^3^Department of Psychology, Sapienza University of Rome, Rome, Italy; ^4^IRCCS Fondazione Santa Lucia, Rome, Italy; ^5^Department of Biotechnological and Applied Clinical Sciences, University of L’Aquila, L’Aquila, Italy

**Keywords:** shift work, sleepiness, psychomotor performance, sustained attention, poor sleep quality

## Abstract

In Europe, 40% of health-care employees are involved in shift work. The altered sleep/wake rhythm of night-shift nurses is also associated with deteriorated cognitive efficiency. In this study, we examine the effects of the night shift on psychomotor performance, sleepiness, and tiredness in a large sample of shift-working nurses and evaluated if poor sleep quality, sex, age, or years on the job could impact on a better adaptation to shift work. Eighty-six nurses with 8-h-rapidly-rotating-shifts were evaluated at the end of three shifts (morning/afternoon/night) for sleepiness and tiredness. Sleepiness, as measured by the Karolinska Sleepiness Scale, and tiredness, as measured by the Tiredness Symptoms Scale, were more pronounced after the night shift. These increases were paralleled by lower attentional performance on the psychomotor vigilance task (PVT) after the night shift. While sex, age, and years on the job did not affect PVT performance after the night shift, lower sleep quality (Pittsburgh Sleep Quality, PSQI > 5) was associated with decreased performance. The high prevalence of altered sleep quality showed that nurses, and shift workers in general, are at risk for a poor sleep quality. The evaluation of sleep quality through PSQI could represent a rapid, inexpensive tool to assess health-care workers assigned to rotating night shifts or to evaluate nurses who coped poorly with night-shift work.

## Introduction

Shift work is increasingly common in developed countries; about 40% of workers in the European Union care industry are involved in shift work (Parent-Thirion et al., 2017). Due to the intrinsic association between sleep regulation and circadian desynchronization, rotating shift workers with night shift experience more sleep/wake cycle disruptions and nodded off more at work more often than did those who worked early day shifts only (Gold et al., 1992). This issue is particularly relevant in the health-care industry, which requires workers to be available in 24-h runs with a rotation of three shifts per day. The altered sleep/wake rhythm of nurses working night shifts is associated with medical errors (Gold et al., 1992; Fabbian et al., 2016; Di Muzio et al., 2019) and, more generally, with their ability to accurately and rapidly attend to basic and advanced attentional performance tasks (Behrens et al., 2019).

Similarly, their ability to perform mental calculations quickly and correctly is impaired compared to those who worked fixed shifts (Molzof et al., 2019). Sleepiness is linked to a higher deterioration of cognitive performance in executive skills and concentration-related procedures among Italian medical specialists who worked on rotating shifts compared to those who worked fixed shifts (Tempesta et al., 2013).

It is crucial to managing shift work so that medical professionals and patients’ safety and health are not negatively affected. Regarding psychological health and performance, in our previous study (Di Muzio et al., 2019), comparing nighttime and daytime psychomotor performance in a small sample of Italian nurses, night shifts are associated with significantly greater sleepiness, tiredness, and worsened performance, such as reduced reaction time to visual cues. In that study, the psychomotor performance was measured by the psychomotor vigilance task (PVT). PVT is considered the most widespread and reliable method to assess the neurocognitive consequences of sleep deprivation and excessive sleepiness on sustained attention (Dinges et al., 1997). Studies utilizing the PVT have observed that at the end of a 12-h shift, nurses appear more vulnerable with decreased response speed and increased sleepiness (Wilson et al., 2019). However, in other studies, even though sleepiness and mental tiredness are more pronounced in night shift workers, none of the negative effects have been replicated in PVT performance (Surani et al., 2015). Thus, more consistent evidence and studies using larger samples are necessary.

The current study aims to extend our previous study by using a much larger sample of nurses and investigating the effects of working 8-h rapidly rotating shifts on tiredness and sleepiness and how they affect psychomotor performance. Moreover, we are also interested in developing criteria to identify nurses with better adaptation to shift work. To this end, we have evaluated how (1) the presence of poor sleep quality, as assessed by the widely used Pittsburgh Sleep Quality Index (PSQI) (Curcio et al., 2013); (2) age, and (3) the amount of experience in terms of years on the job may modulate the hypothesized effect of night shifts in worsening psychomotor performance.

## Methods

### Participants

Eighty-six nurses (55 females; 31 males) working an 8-h rapidly rotating forward shift participated in the study. They were recruited from the Policlinico Umberto I (*N* = 48), the San Giovanni Addolorata (*N* = 20), and the Sant’Andrea (*N* = 18) hospitals in Rome.

As shown in Table 1, age and years of work were significantly different between sexes. Specifically, males were older and had more years of work experience than females. For this reason, data of the current study were analyzed by controlling for sex differences (see section “Data analysis”).

**TABLE 1:** Demographic and Pittsburgh Sleep Quality Index (PSQI) variables.

	Shift			
	Females (*N* = 55)	Males (*N* = 31)	t_84_	*p*
Age	37.8 (1.20)	42.2 (1.87)	–2.07	0.04
Years on the job	12.5 (1.10)	16.6 (1.52)	–2.19	0.03
**PSQI**				
Sleep duration	1.15 (0.08)	1.23 (0.14)	–0.54	0.59
Sleep disturbances	1.25 (0.12)	1.39 (0.19)	–0.61	0.55
Sleep latency	1.00 (0.11)	1.16 (0.16)	–0.85	0.40
Daytime dysfunctions	0.45 (0.11)	0.52 (0.17)	–0.31	0.76
Sleep efficiency	1.18 (0.07)	1.29 (0.08)	–0.98	0.33
Sleep quality	0.20 (0.09)	0.10 (0.10)	0.75	0.45
Medications to sleep	1.04 (0.11)	0.90 (0.12)	0.78	0.44
Total score	6.29 (0.41)	6.58 (0.56)	–0.42	0.68

*Means (SE) of demographic variables and components of the PSQI as a function of Sex of participants.*

As inclusion criteria, we considered:

• No medical and chronic disorders as assessed with a clinical interview.
• Adherence to a collection of the measurement data according to the following sequence of shifts: morning (M) – afternoon (A) – night (N). No change of shift was allowed for the 3 days under investigation.
• No nap taken during the night shift.
• Trainee nurses were excluded from the study.

Each participant signed an informed consent form before the study enrolment. Participation was anonymous and voluntary. The protocol was approved by the Ethical Committee of Sapienza University of Rome (# 343/17 on 26/04/2017).

### Measures

The PSQI is a self-assessment questionnaire consisting of nine items and provides a reliable, valid, and standardized sleep quality measurement (Italian validation) (Curcio et al., 2013). The PQSI assesses sleep habits over 1 month before the evaluation. This self-reported questionnaire includes 19 items divided into 7 subscales that assess sleep quality, sleep latency, sleep duration, habitual sleep efficiency, sleep disorders, the use of sedative-hypnotics, and complaints during the day. The sum of the scores of the seven components gives the overall score, which has a range between 0 and 21, with “0” indicating the absence of difficulty and “21” severe difficulties in all areas. Scores above 5 indicate poor sleep quality.

The Karolinska Sleepiness Scale (KSS) (Italian adaptation) (Turco et al., 2015) is a 9-graded scale to evaluate the subjective level of sleepiness. Considering that 1 and 9 indicate a state of minimal and maximal sleepiness, the participant must report the level that best reflects his/her psychophysical state in the 5 min before administering the questionnaire.

The Tiredness Symptoms Scale (TSS) (Italian adaptation) (Palagini et al., 2016) is a 14-item questionnaire assessing tiredness’s physical and emotional symptoms at the time of evaluation. Participants indicated the presence/absence of the symptoms indicated in the list. The final score is the sum of the positive responses related to the symptoms.

The PVT is a well-established computerized simple cued reaction time task that provides the most widely used metrics of sustained attention (Dinges and Powell, 1985), very sensitive to the effects of sleep loss (Dinges et al., 1997). During the PVT, participants are in front of a computer screen for 5 min. They were asked to click the left mouse button every time a number appears at irregular intervals. The counter started to scroll at random intervals, and participants had to stop it as quickly as possible by left-clicking on the computer mouse. Even a half-second of delay in response (i.e., lapses) may be evidence of microsleep. The task contained 60 trials.

### Procedure

At the end of the work shift, each participant was interviewed: morning (7:00–13:30), afternoon (13:30–20:00), and night (20:00–7:00). Their roster was as follows: day A – Morning; day B – Afternoon; day C – Night; day D – Post-night shift day; day E – Rest. After day E, the sequence started again. Hence, all nurses were evaluated at the end of the shift and for three consecutive days, starting from day A to day C. Each participant was invited to complete our measurement protocol (KSS, TSS, and PVT) at the end of the work shift. The PSQI was administered only at the end of the first day/shift (i.e., the morning shift). The maximum completion time was 15 min. All sessions took place in a room without noise and environmental distractions. Nurses were asked to switch off their mobile phones during the session, and no person was allowed entry except the experimenter.

The questionnaires were administered in the following fixed order: (a) KSS, (b) TSS, and (c) PVT. The same sequence was replicated at the end of each of the three shifts investigated.

### Data Analysis

The following dependent variables were considered: TSS and KSS scores, and measures of the PVT [median reaction time (RT), speed (reciprocal of RT), lapses (>500-msec RT), 10% slowest RT, 10% fastest RT, and response time divergence (RTD), which represents a measure of variability (i.e., dissimilarity of probability density functions of RT)]. Each variable was analyzed using a mixed design Sex x Shift (M vs. A vs. N) Analysis of Covariance (ANCOVA), with age and years on the job as covariates. Paired *t*-tests were used for the *post hoc* comparisons between the three means. The significance level was set at *p* < 0.05.

## Results

Female and male nurses did not differ on any sleep quality dimension, as assessed by the PSQI subscales and by the corresponding total score (Table 1). Notably, the average total score exceeded the > 5 cutoff for this test. According to this cutoff, 47/86 participants (54.6%) have poor sleep quality.

**FIGURE 1:**
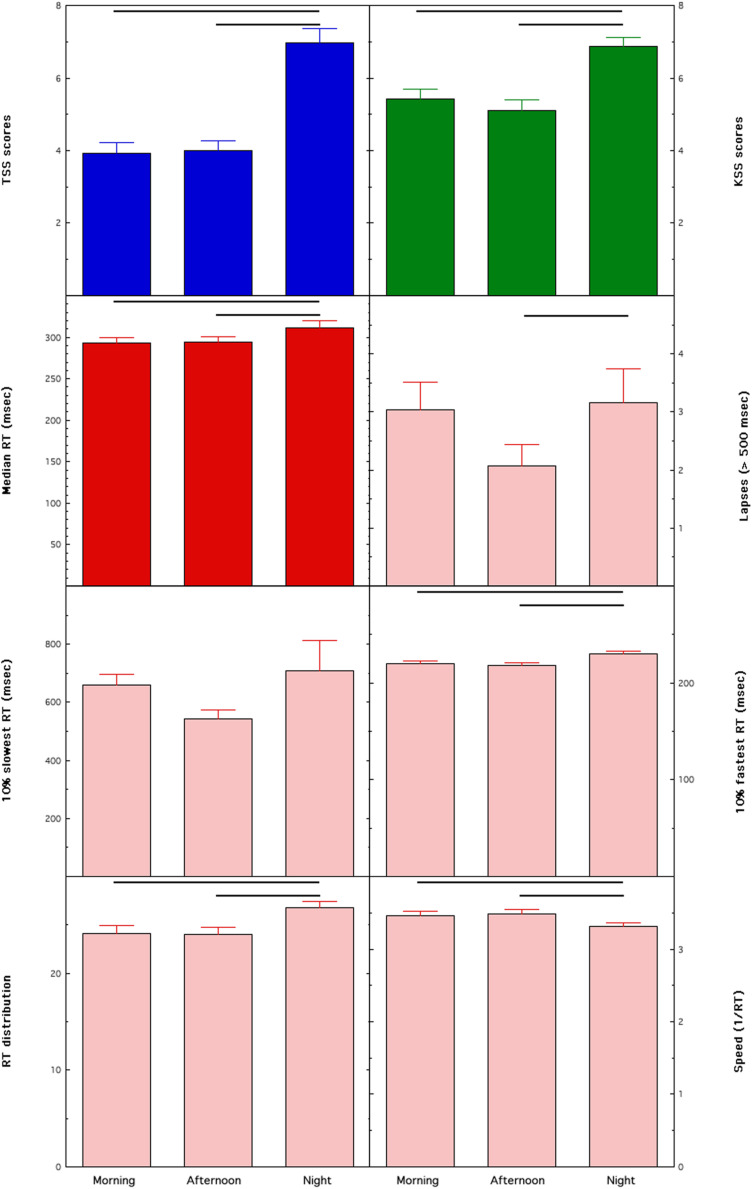
Means and standard errors of the dependent measures collected in female and male nurses across different rapidly rotating shifts (morning, afternoon, and night). Tiredness Symptoms Scale (TSS), Karolinska Sleepiness Scale (KSS), medians of Reaction Times (RT) on the Psychomotor Vigilance Task (PVT) (median RT). Horizontal lines highlight significant comparisons between conditions.

Table 2 reports the results of the ANCOVAs on the dependent measures of the study. The main effect of Shift was significant for each variable, except for 10% slowest RT. The main effect of Sex was significant only for some PVT measures (median RT, 10% fastest RT, RT distribution, and speed). In all cases, differences point to better performance of males. No Sex × Shift interaction was significant, except the variable Speed of the PVT. The effects of the two covariates, Age and Years on the job, were never significant. These effects are shown in Figure 1.

**TABLE 2:** Results of the statistical analyses on the dependent measures of the study.

	Sex		Shift		Sex x Shift		Multivariate tests Within-cell regression (Age and Years on the job, as covariates)	
	
	*F* (1,82)	*p*	*F* (2,168)	*p*	*F* (2,168)	*p*	Wilk’s Lambda Rao R (6,160)	*p*
TSS	2.33	0.13	**59.50**	**<0.00000001**	0.15	0.86	0.97 0.38	0.89
KSS	0.38	0.53	**18.37**	**0.0000001**	0.66	0.52	0.86 2.04	0.06
**PVT**								
Median RT	**4.49**	**0.04**	**5.34**	**0.006**	1.82	0.16	0.97 0.41	0.87
10% slowest RT	1.96	0.16	1.87	0.16	0.18	0.83	0.99 0.13	0.99
10% fastest RT	**4.11**	**0.05**	**8.32**	**0.0003**	2.20	0.11	0.91 1.28	0.27
Minor lapses (<500 msec)	0.88	0.35	**2.97**	**0.05**	1.45	0.24	0.95 0.65	0.69
RT distribution (RTD)	**4.65**	**0.04**	**9.27**	**0.0001**	2.10	0.13	0.92 1.16	0.32
Speed	**6.71**	**0.01**	**8.61**	**0.0003**	**3.61**	**0.03**	0.96 0.54	0.78

*Results of the Sex × Shift (Morning, Afternoon, Night) ANCOVAs on the dependent measures [Tiredness Symptoms Scale (TSS), Karolinska Sleepiness Scale (KSS), variables of the Psychomotor Vigilance Task (PVT)], and considering Age and Years on the job as covariates. Bold values denote statistical significance at the *p*≤0.05 level.*

Tiredness, as measured by the TSS, had a significant effect in the direction of greater tiredness after the N shift compared to the M (*p* < 0.00000001 at the *post hoc t*-test) and A shift (*p* < 0.00000001), while the M and A shifts were not significantly different (*p* = 0.69). Similarly, sleepiness, as measured by the KSS, had significantly higher values after the N shift compared to the M (*p* = 0.000003) and A shift (*p* < 0.00000001), while the M and A shifts were not significantly different (*p* = 0.26).

The median RTs were significantly higher after the N shift compared to the M (*p* = 0.002) and A shift (*p* = 0.01), while the M and A shifts were not different (*p* = 0.55). A similar pattern of differences was found for the lapses (N > A, *p* = 0.01; N vs. M *p* = 0.46; M vs. A *p* = 0.08), the 10% fastest RT (N > M, *p* = 0.0003; N > A, *p* = 0.0003; M vs. A, *p* = 0.98), the RTD values (N > M, *p* = 0.00007; N > A, *p* = 0.0003; M vs. A, *p* = 0.67), and the Speed values (N < M, *p* = 0.0002; N < A, *p* = 0.0003; M vs. A, *p* = 0.88).

**FIGURE 2:**
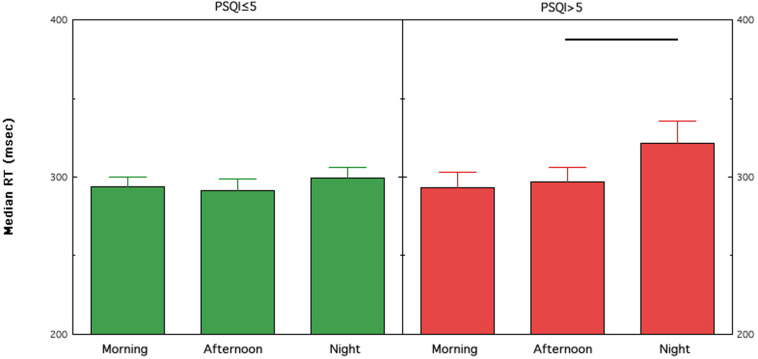
Means and standard errors of the medians of RTs on the PVT (PVT; median RT) collected in female and male nurses with (PSQI > 5) or without (PSQI ≤ 5) poor sleep quality across different rapidly rotating shifts (morning, afternoon, and night). Horizontal lines highlight significant comparisons between conditions. PSQI, Pittsburgh Sleep Quality Index.

According to the aim of assessing the moderating effect of the presence of poor sleep quality on the behavioral consequences of shift work, we compared the primary variable of the PVT (i.e., median RT) in two groups: those with (PSQI > 5) and without sleep problems (PSQI ≤ 5). A significant effect for Shift was present in nurses with sleep problems [*F*(2,90 = 3.79; *p* = 0.03)] and was not significant in nurses without sleep problems [*F*(2,90 = 0.78; *p* = 0.46)]. No other main effect or interaction was significant for both groups. Figure 2 shows that the significant effect in nurses with sleep problems is explained by worse performance in the N shift compared to the A shift (*p* = 0.03), a close to significant difference compared to the M shift (*p* = 0.09), while the M vs. A comparison was not significant (*p* = 0.45).

## Discussion

Shift work is reportedly associated with higher risks in quality of care and safety among services provided by the health-care sector (Barger et al., 2009), and the night shifts increase sleepiness and reduce psychomotor performance in hospital staff (Ganesan et al., 2019). Furthermore, night shifts in nurses also affect other dimensions of attentional processing, like selective attention (Niu et al., 2013). Identifying the risk that rapidly rotating shift working may pose to sleep quality is possible to establish procedures designed to minimize errors in the intensive working practice of health-care services and hospitals (Folkard and Lombardi, 2006).

This study confirms previous findings using the PVT (Di Muzio et al., 2019; Ganesan et al., 2019), and describe the impact of shift work in a large sample of nurses. Specifically, nurses working rapidly rotating shifts experienced increased sleepiness and tiredness immediately at the end of the night shift compared to daytime shifts. Associated with the effects of sleepiness and tiredness, the performance on the PVT is deteriorated in nurses assigned to rotating night shift compared to the day shifts. We have carefully controlled for possible variables on alertness and performance remain impaired during night shifts. Crucially, only nurses with poor sleep quality (>5 scores at the PSQI) show worsening of sustained attention after the night shift. No such relationship on PVT performance was observed for sex, age, and shift experience in terms of years on the job. Notably, the effect of poor sleep quality does not affect the “baseline” performance (i.e., after the morning shift), since the two groups show very similar performance (PSQI ≤ 5 = 293.74 ± 6.44 msec, PSQI > 5 = 293.39 ± 9.82 msec; t_84_ = 0.03, *p* = 0.98).

Although several studies have reported increased sleepiness and lower performance associated with the night shift, the strength of the current study is represented by (A) the sample size [i.e., 80 participants in a within-subject study], (B) the behavioral assessment of vigilance and (C) the investigation on some moderating factors. The behavioral assessment is crucial since self-rated alertness sometimes does not coincide with healthcare workers’ objective performance (Ganesan et al., 2019). Furthermore, the behavioral assessment could help predicting performance errors. Although, to the best of our knowledge, there are no studies directly evaluating the association between performance at the PVT and medical errors yet, indeed, some studies report that reduced sustained attention predicts errors in workplaces (e.g., Edkins and Pollock, 1997; Basner and Rubinstein, 2011). Hence, we can hypothesize that the worsened sustained attention associated to night shifts could help prediction of errors in health care, and its empirical evaluation should be in the research agenda.

However, we have to mention that this is a field study that cannot control a possible confound of prior shifts worked and of time spent awake. This intrinsic confound should be considered as a limitation to the study. Some accumulation of sleepiness and lower PVT performance after the night shift could also be partially due to consecutive shifts worked, not just the night shift itself. Also, time of day should be considered as a possible confound, since the worsened performance at the end of the night shift may be affected by reduced sleep across the night, extended wakefulness, particularly if nurses have stayed awake for the entire day prior to the night shift, and the circadian timing of the test. For example, at the end of the first night shift, staff are likely to have been awake for longer than the end of a morning shift if they did not nap prior to the shift, and to have had less sleep in the prior 24 h. Further to these factors, however, the timing of the testing at the end of the night shift occurs in the early morning (7:00), likely to still be associated with a circadian drive for sleepiness for some individuals at least. Compared to testing at the end of the afternoon shift, for example, which occurs at 20:00, timing that nears the Wake Maintenance Zone and thus associated with lower sleepiness (Ganesan et al., 2019).

Taken together, our data suggest that rapid rotation shift increases sleepiness and reduces psychomotor performance, but poor sleep quality exacerbates behavioral consequences of night shift work in nurses. In particular, more than half of nurses with poor sleep quality experienced degradation of attention due to shift work. This finding confirms that shift workers (Kecklund and Axelsson, 2016), more generally, and nurses working in rotating shifts (Sun et al., 2019), in particular, are at high risk for sleep disruption/poor sleep quality. Misaligned endogenous circadian rhythm and forced sleep timing may lead to sleep fragmentation, and sleep/wake disturbances interact to affect alertness and performance with essential health and safety implications.

However, the association between poor sleep quality and exacerbation of behavioral consequences of night shift is intrinsically correlational, and the current study does not point to a causal relationship. It is also possible that other factors make people more vulnerable to the consequences of night shifts, and these consequences, in turn, affect sleep quality. The current study suggests that sex, age, and years on the job have a substantial contribution on this relationship.

Although a causal relationship cannot be invoked, the large proportion of nurses with poor sleep quality (55% in the current study) is coherent with studies in large populations of shift workers (Kim et al., 2015). Therefore, this study may be clinically important to further study the links between shift work status and sleep quality and to identify a possible and viable approach to address this serious problem for health and care workers. In our previous study, we suggested monitoring of sleep-wake schedules and, in general, of medical personnel using actigraphs (Smith et al., 2018) or specific and valid sleep trackers (e.g., de Zambotti et al., 2019) to track their sleep-wake schedules; the final aim of such tracking is to trigger appropriate countermeasures when a poor sleep quality or an altered sleep-wake schedule is suspected. Here, we suggest using an inexpensive and simple test like the PSQI as a screening tool for nurses to assign to the night shifts. This screening test should lead to a proper clinical evaluation in cases of scores indicating poor sleep quality. Even more, it can be used as a periodic check of a nurse’s ability to cope with the night shift.

We are aware that this does not solve the problem on a large scale, and appropriate intervention strategies are needed in hospitals to improve sleep quality among shift-work nurses. Applied research is developing adequate countermeasures for all people who work shifts in health care. For example, a prophylactic nap during the night shift improves executive functions and could maintain concentration-related skills (Tempesta et al., 2013). This strategy, however, seems more appropriate for shifts longer than 8 h. Indeed, policy changes in nursing practice should consider effective treatments for reducing sleepiness associated with night shifts. For this reason, we plan to assess the efficacy of exposure to bright light (Huang et al., 2013) at the start of the night shift for reducing sleepiness to the levels of morning and afternoon shifts.

## Data Availability Statement

The raw data supporting the conclusions of this article will be made available by the authors, without undue reservation.

## Ethics Statement

The studies involving human participants were reviewed and approved by Ethical Committee of Sapienza University of Rome (# 343/17 on 26/04/2017). The patients/participants provided their written informed consent to participate in this study.

## Author Contributions

LD, MD, AG, and MP substantial contributions to the conception and design of the work. LN, GD, ED, FS, and LA acquisition and analysis of data. MD, AG, LD, LN, and VA interpretation of data. MD, AG, LD, VA, LN, SS, and MP drafting the work and revising it critically for important intellectual content. MD, VA, SS, AG, LD, LN, GD, ED, LA, FS, and MP final approval of the version to be published. MD, MP, AG, LD, VA, SS, LN, GD, ED, LA, and FS agreement to be accountable for all aspects of the work in ensuring that questions related to the accuracy or integrity of any part of the work are appropriately investigated and resolved. All authors contributed to the article and approved the submitted version.

## Conflict of Interest

The authors declare that the research was conducted in the absence of any commercial or financial relationships that could be construed as a potential conflict of interest.
